# Integrated Analyses of Microbiomics and Metabolomics Explore the Effect of Gut Microbiota Transplantation on Diabetes-Associated Cognitive Decline in Zucker Diabetic Fatty Rats

**DOI:** 10.3389/fnagi.2022.913002

**Published:** 2022-06-03

**Authors:** Tingting Bi, Lijing Zhang, Libin Zhan, Ruiqi Feng, Tian Zhao, Weiming Ren, Tianyi Hang, Wen Zhou, Xiaoguang Lu

**Affiliations:** ^1^School of Traditional Chinese Medicine and School of Integrated Chinese and Western Medicine, Nanjing University of Chinese Medicine, Nanjing, China; ^2^Center for Innovative Engineering Technology in Traditional Chinese Medicine, Liaoning University of Traditional Chinese Medicine, Shenyang, China; ^3^Key Laboratory of Ministry of Education for TCM Viscera-State Theory and Applications, Liaoning University of Traditional Chinese Medicine, Shenyang, China; ^4^Department of Emergency Medicine, Zhongshan Hospital, Dalian University, Dalian, China

**Keywords:** diabetes-associated cognitive decline, gut microbiota transplantation, microbiota-gut-brain axis, insulin and leptin resistance, β-amyloid

## Abstract

Diabetes-associated cognitive decline (DACD), one of the complications of type 2 diabetes (T2DM), correlates significantly with the disorder in glycolipid metabolism, insulin/leptin resistance, and accumulation of β-amyloid (Aβ). Although gut microbiota transplantation (GMT), a novel non-invasive physiotherapy strategy, has been a promising intervention to alleviate the symptoms of T2DM, its protective effect on progressive cognitive decline remains elusive. Here, we transplanted the gut microbiota of healthy or cognitive decline donor rats into ZDF or LZ rats, and integrated microbiomics and metabolomics to evaluate the directional effect of the gut microbiota on the recipient rats. The basal metabolism phenotype changed in ZDF rats instead of in LZ rats. One possible mechanism is that the microbiota and metabolites alter the structure of the intestinal tract, stimulate the brain insulin and leptin signaling pathways, and regulate the deposition of Aβ in the brain. It is worth noting that 10 species of genera, such as *Parabacteroides*, *Blautia*, and *Lactobacillus*, can regulate 20 kinds of metabolites, such as propanoic acid, acetic acid, and citramalic acid, and having a significant improvement on the cognitive behavior of ZDF rats. In addition, the correlation analysis indicated the gut microbiota and metabolites are highly associated with host phenotypes affected by GMT. In summary, our study indicates that altering the microbiota-gut-brain axis by reshaping the composition of gut microbiota is a viable strategy that has great potential for improving cognitive function and combatting DACD.

## Introduction

The global prevalence of type 2 diabetes mellitus (T2DM) has increased over the past two decades, and diabetes-associated cognitive decline (DACD) is a severe neurologic disorder that impairs memory of T2DM (Riederer et al., 2017; Srikanth et al., 2020). Previous studies have found that DACD is associated with cerebral insulin resistance, excessive deposition of β-amyloid (Aβ) in the brain, neurotransmitter metabolism disturbance, and mitochondrial dysfunction (Biessels and Despa, 2018). Recent studies also have emphasized the critical impact of the gut microbiota on regulating insulin resistance and leptin resistance via the generation of functional microbial metabolites in T2DM animal models and humans (Zhu et al., 2019). Moreover, the gut microbiota plays a vital role in linking host cognitive function and Aβ pathology (Bo et al., 2020). Microbiota homeostasis is essential for the maintenance of host metabolic homeostasis and for modulating host cognitive function via the regulation of Aβ brain clearance (Mezo et al., 2020; Sun M. et al., 2020).

Re-structuring of the gut microbiota can alter the host cognitive function. Studies on germ-free (GF) and antibiotic (ABX)-treated animals have shown that gut microbiota transplantation is associated (at least in part) with alterations in cognitive behavior, brain function, and neuropathological characteristics (Zhan et al., 2018; Uzbay, 2019; Yu et al., 2019). Animals provided with specific bacterial strains or gut microbiota exhibit altered behavior, and human studies of such strains have confirmed the potential translatability of such findings (Kim et al., 2020; Ding et al., 2021; Salehi et al., 2021). However, the functional redundancy in the microbiome makes it difficult to attribute the causal effects of the diverse taxa in the disease. Therefore, it is necessary to go beyond the characterization of intestinal microbial composition and analyze the functional output of the microbiome (i.e., metabolome) in order to have a more comprehensive understanding of the role of different microbial classifications in diseases. Recent analyses using integrated analyses of microbiomics and metabolomics found that changes in the gut microbiota can improve T2DM metabolic profiles, counteracting dysfunction of glycolipid metabolism and insulin/leptin resistance (Wang et al., 2018; Shao et al., 2020; Li et al., 2021). Alterations in the gut microbiota composition have been achieved by transplanting gut microbiota from normal animals or humans, and colonization is achieved in GF or ABX-treated animals (Cani, 2019; Liu et al., 2020). These findings provide evidence for a remote relationship between the intestine and the brain, suggesting that colonization to introduce a novel gut microbiota into the host might represent a potential therapeutic modality for DACD.

In this study, we examined the effect of the gut microbiota on the pathogenesis of DACD in a spontaneous T2DM model. Zucker Diabetic Fatty (ZDF) rat is an obese rat model with leptin receptor deficiency and animals of this strain become a stable T2DM model at 9 weeks of age, exhibiting increased blood glucose levels when maintained on an induction diet LabDiet 5008 (Yin et al., 2018; Zhou et al., 2019). We observed the emergence of cognitive impairment complications and associated alterations in the composition of the gut microbiota community in 15-week-old ZDF rats (compared with the lean control LZ rats), and then gut microbiota transplantation (GMT) were performed. Our study aims to clarify the role of GMT through mediating microbiota-gut-brain to alter the cognitive function, thereby highlight the potential utility of re-structuring the core gut microbiota as an effective intervention for metabolism-implicated neurological diseases.

## Materials and Methods

### Animals

All rats were purchased from Beijing Vital River Laboratory Animal Technology (VRL; Beijing, China) and housed under the specific pathogen-free (SPF) conditions (temperature, 23 ± 2°C; relative humidity, 65% ± 5%; 12 h/12 h light/dark cycle, 07:00–19:00) with free access to food and water. Animals were housed in the animal experiment center at Nanjing University of Chinese Medicine. LZ rats were fed standard chow (MD17121, Medicience, China), while ZDF rats were provided a diabetogenic diet of Formulab chow (LabDiet 5008; Purina, United States). All animal procedures were performed at Nanjing University of Chinese Medicine (Nanjing, China) in accordance with the National Institutes of Health Guide for the Care and Use of Laboratory Animals and a study-specific animal protocol that was approved by the Animal Ethics Committee of Nanjing University of Chinese Medicine (Approval No. 201812A009).

### Preparation of Donor Gut Microbiota

15-week-old male ZDF (*fa/fa*) rats (396.55 ± 33.94 g, Z group) and their lean control 15-week-old male LZ (*fa/* +) rats (302.97 ± 14.28 g, L group) were used as donors, and cecal/colon contents were collected after isoflurane anesthesia. An aliquot (0.1 g) of each cecal/colon content sample was collected for use in 16S rRNA analysis and the remainder of each sample was diluted 1:20 in sterile phosphate-buffered saline (PBS) and centrifuged at 188 × g for 5 min. The supernatant from each sample was filtered through a 70-μm filter, and then combined and aliquoted.

### Antibiotic Administration and Gut Microbiota Transplantation

**FIGURE 1:**
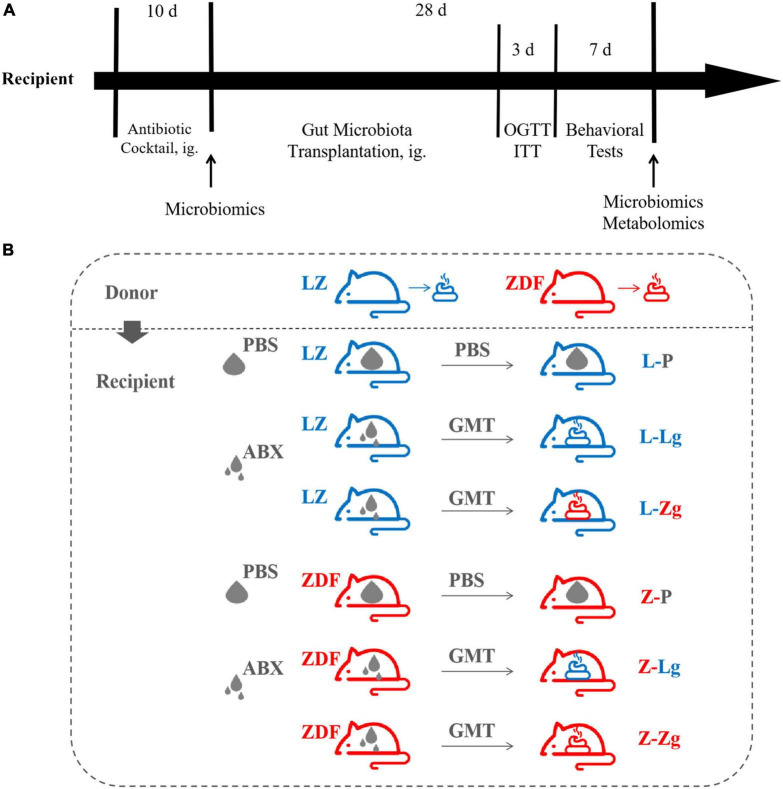
Experimental design of gut microbiota transplantation (GMT). **(A)** Timeline of experiment. **(B)** Different treatment groups of experiment.

Recipient 9-week-old male LZ rats (221.95 ± 11.55 g) and 9-week-old male ZDF rats (282.37 ± 15.98 g) were allowed to acclimatize to the environment for 3 days and divided into 6 groups, respectively: L-P group, L-Lg group, L-Zg group, Z-P group, Z-Lg group, Z-Zg group. Antibiotic administration and GMT were performed according to the method described previously (Zhang et al., 2020). Briefly, rats in need of gut microbiota transplantation (L-Lg group, L-Zg group, Z-Lg group, Z-Zg group) were gavaged with 1 mL/rat/day of a broad-spectrum antibiotic mixture (ampicillin, gentamicin, metronidazole, neomycin, each 0.25 mg/mL) for 10 consecutive days; PBS-treated rats (L-P group and Z-P group) were gavaged with 1 mL/rat/day of PBS buffer instead of antibiotic mixture for 10 consecutive days. After the last antibiotic treatment, the rats in need of gut microbiota transplantation were given the corresponding donor gut microbiota by gavage with 750 μL/rats/day for 28 consecutive days; PBS-treated rats were continued to be given 750 μL/rats/day by gavage with PBS for 28 consecutive days. Fresh fecal samples were collected from the recipients before the initiation of the GMT procedure, and cecal/colon contents were collected from euthanized animals at the end of the experiment. The different treatment groups involved in the study and the experimental timeline of GMT and post-GMT parameters in recipient rats are shown in Figure 1.

### Novel Object Recognition Test

The rats were habituated in the experimental environment for 1 h prior to test, and then placed in the open field freely for 10 min. Two objects named “object” used for discrimination in the first day of training were green cylinder (object A) and red cone (object B), which were of different shapes and colors. The interval time between first and second exposures for short- and long- term memories working were 1 and 24 h, respectively. Rats were returned to the open field to investigate two objects, which object B was replaced by blue cuboid (object C). Exploration of an object was defined as directing the nose to object at distance of less than 2 cm. Data was recorded by the camera and the discrimination index was expressed according to the formula: Discrimination Index = t_*C*_/(t_*C*_ + t_*A*_) × 100%. Wipe the open field with 75% ethanol in order to get rid of olfactory cues between each trial.

### Morris Water Maze Test

Spatial learning and memory were evaluated by the MWM tests, as described previously (Bi et al., 2021). The MWM tests (the orientation navigation test and the spatial exploration test) were preceded by 5 days. Briefly, for four consecutive days in the orientation navigation test, rats were trained with four trials per day and each trial consisted of allowing the animals to swim for up to 120 s, until the rats reached the submerged platform. Between the trials on a given day, each rat was allowed to rest for 60 s. If the rat failed to reach the platform within 120 s, the animal was guided gently to the platform and allowed to rest for 60 s. The platform was removedafter the orientation navigation test. In the spatial exploration test, rats had to swim for 120 s and the percentage of time in each quadrant, crossing number the original of platform location, and time in searching original platform location were recorded by an automated analysis system (TopScan, United States).

### 16S rRNA Sequence Analysis

Genomic DNA from fecal and cecal/colon contents was extracted using the Fast DNA SPIN extraction kit (MP Biomedicals, Santa Ana, CA, United States). DNA quantity and quality were determined using a NanoDrop ND-1,000 spectrophotometer (Thermo Fisher Scientific, Waltham, MA, United States). The hypervariable regions of the 16S rRNA gene (V3-V4 region) were amplified, and sequencing data were analyzed using the Quantitative Analysis of Microbial Ecology software (QIIME, v1.8.0). The resulting data then were organized into OTUs based on 97% similarity using QIIME software. The datasets generated in this study have been deposited in the NCBI Sequence Read Archive (SRA) database (Accession Number: SRP301360).

### Targeted Metabolomic Analysis

To extract metabolites, each cecal/colon content sample was combined with ultrapure water, zirconium oxide beads, and acetonitrile/methanol. The mixture was homogenized and centrifuged. The resulting supernatant was subjected to automatic derivatization and separation of using a Biomek 4,000 Workstation (Beckman Coulter, Inc., United States). Microbial metabolites were analyzed in a random injection sequence by an UPLC-MS/MS system (ACQUITY UPLC-Xevo TQ-S, Waters Corp., United States). To ensure optimal instrument performance and the consistent high quality of analytical results, a comprehensive set of rigorous quality control/assurance procedures was employed. Data preprocessing was performed using QuanMET (v.2.0, Metabo-Profile, Shanghai, China). Metabolite identification was based on accurate mass and product ion spectra matching with online databases and literature.

### Basal Metabolic Determinations

Body weight (BW), abdominal circumference (AC), and random blood glucose (RBG) levels of each rat were measured weekly throughout the experiment. Oral glucose tolerance test (OGTT) was performed by fasting animals overnight (approximately 14 h) and then administering glucose (2 g/kg body weight) by oral gavage. Insulin secretion test (ITT) was performed by fasting animals for 6 h and then administering the animals with insulin (0.5 U/kg body weight) by intraperitoneal injection. For both OGTT and ITT, glucose levels in tail vein blood were measured at the indicated time points using a OneTouch glucometer (Roche DC Japan K.K., Tokyo, Japan). The resulting values were used to calculate the area under the curve (AUC). Additionally, terminal blood samples were collected from each rat at the time of euthanasia. Serum triglyceride (TG), total cholesterol (TC), low-density lipoprotein cholesterol (LDL-C), high-density lipid cholesterol (HDL-C), and glycosylated hemoglobin (HbA1c%) were measured by an automated biochemical analyzer. Fasting serum insulin levels (FSI; Mercodia, Sweden) and leptin levels (R & D Systems, United States) were determined in strict accordance with the experimental procedure. To evaluate insulin resistance, the homeostasis model of assessment for insulin resistance index (HOMA-IR) was calculated using the following formula: HOMA-IR = fasting blood glucose (FBG) (mmol/L) × FSI (mIU/L)/22.5.

### Western Blotting

Each brain sample was homogenized in RIPA lysis buffer (Beyotime, China) containing 1% Protease Inhibitor Cocktail (Cell Signaling Technology, United States) and 1% Phosphatase Inhibitor Cocktail (Cell Signaling Technology, United States). The total protein of the lysates was separated by 8% sodium dodecyl sulfate-polyacrylamide gel electrophoresis (SDS-PAGE) and transferred to a polyvinylidene fluoride (PVDF) membrane (Millipore, Billerica, MA, United States). Expression levels of target proteins were detected and relative densities of the bands were analyzed using the ImageQuant TL 1D system (GE Healthcare, United States).

### Congo Red Staining

The whole brain of each rat was fixed in 4% paraformaldehyde (Solarbio, China) for 24 h, and dehydrated by passaging through a graded series of sucrose solutions. The brain then was embedded by ice-chiled OCT gel, and coronal sections from the hippocampus and cortex were prepared at 20 μm thick. For labeling amyloid deposits, brain slices were stained with 1% Congo red solution (Sigma-Aldrich, St. Louis, MO, United States) in 80% of absolute ethanol and 1% of NaOH. After being washed, sections were counterstained with cresyl violet, dehydrated in absolute ethanol, and then cleared in xylene. Specimens were mounted on slides and evaluated under a light microscope (OLYMPUS, Tokyo, Japan) with the magnification of 40 ×.

### Analysis of Aβ42 and Aβ40 Levels by Enzyme-Linked Immunosorbent Assays

Brain hippocampus and cortex samples were combined with Tris-saline buffer (150 mM NaCl, 50 mM Tris HCl, pH 7.6) in the presence of PMSF (Beyotime, China), and the mixture was homogenized and extracted. The resulting supernatants were collected after centrifugation and soluble Aβ42 and Aβ40 levels were measured using ELISA kits (Wako, Japan) according to the manufacturer’s instructions. The absorbance of each well was read at a wavelength of 450 nm using a microplate spectrophotometer. Meanwhile, the pellets recovered from the extraction step were resuspended by homogenization in 70% formic acid. These homogenates were centrifuged, and the resulting supernatants were neutralized by 20-fold dilution in 1 M Tris Base (pH 11.0). The levels of pellet-derived (insoluble) Aβ42 and Aβ40 then were measured using the same ELISA kits and methods as above.

### Statistical Analysis

The data used for basal metabolic analysis and behavioral analysis are expressed as mean ± standard deviation (mean ± SD). Differences between data from 2 groups were analyzed by two-tailed unpaired Student’s *t*-test. Differences among more groups were analyzed by two-tailed two-way ANOVA with *post hoc* Tukey’s multiple comparison tests where indicated. Analyses were performed using Prism software (v. 8.0; GraphPad, San Diego, CA, United States).

## Results

### Gut Microbiota Transplantation Changes Glycolipid Metabolism Disorder and Cognitive Impairment

**FIGURE 2:**
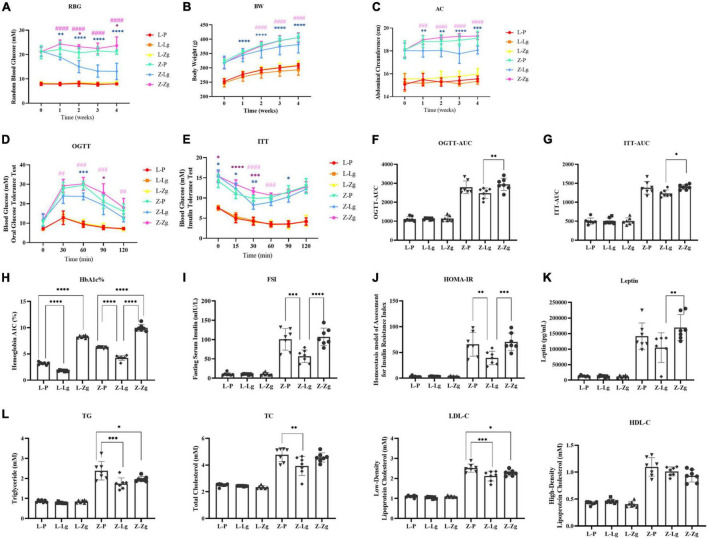
GMT changes glycolipid metabolism disorder in ZDF recipient rats. **(A)** Random blood glucose [RBG; Time: *F*_(4_, _24)_ = 5.870, *P* = 0.0019; Group: *F*_(5_, _30)_ = 423.5, *P* < 0.0001]. **(B)** Body weight [BW; Time: F_(4_, _24)_ = 983.0, *P* < 0.0001; Group: *F*_(5_, _30)_ = 57.03, *P* < 0.0001]. **(C)** Abdominal circumference [AC; Time: *F*_(4_, _24)_ = 16.38, *P* < 0.0001; Group: *F*_(5_, _30)_ = 176.0, *P* < 0.0001]. **(D)** Oral glucose tolerance test [OGTT; Time: *F*_(4_, _144)_ = 195.8, *P* < 0.0001; Group: *F*_(5_, _36)_ = 96.97, *P* < 0.0001]. **(E)** Insulin secretion test [ITT; Time: *F*_(5_, _30)_ = 109.6, *P* < 0.0001; Group: *F*_(5_, _30)_ = 182.5, *P* < 0.0001]. **(F)** Area under the curve of the oral glucose tolerance test [OGTT-AUC; *F*_(5_, _36)_ = 100.2, *P* < 0.0001]. **(G)** Area under the curve of the insulin secretion test [ITT-AUC; *F*_(5_, _36)_ = 144.9, *P* < 0.0001]. **(H)** Glycosylated hemoglobin [HbA1c%; *F*_(5_, _36)_ = 506.3, *P* < 0.0001]. **(I)** Fasting serum insulin [FSI; *F*_(5_, _36)_ = 55.61, *P* < 0.0001]. **(J)** Homeostasis model of assessment for insulin resistance index [HOMA-IR; *F*_(5_, _36)_ = 43.14, *P* < 0.0001]. **(K)** Leptin [*F*_(5_, _3)_ = 38.58, *P* < 0.0001]. **(L)** Triglyceride [TG; *F*_(5_, _36_ = 62.40, *P* < 0.0001], total cholesterol [TC; *F*_(5_, _36)_ = 57.76, *P* < 0.0001], low-density lipoprotein cholesterol [LDL-C; *F*_(5_, _36)_ = 150.3, *P* < 0.0001] and high-density lipid cholesterol [HDL-C; *F*_(5_, _36)_ = 80.15, *P* < 0.0001]. Data are shown as mean ± SD (*n* = 7 per group). *P < 0.05, ^**^P < 0.01, ^***^P <0.001, ^****^P < 0.0001, compared with Z-P group; ^##^*P* < 0.01, ^###^*P* < 0.001, ^####^*P* < 0.0001, compared with Z-Lg group in **(A–E)**. *P < 0.05, ^**^P < 0.01, ^***^P < 0.001, ^****^P < 0.0001 in **(F–L)**. One-way or two-way ANOVA, followed by Tukey’s *post-hoc* test.

To clarify the key role of the gut microbiota in mediating the pathogenesis of DACD, we pretreated the recipient rats with a broad-spectrum ABX cocktail to eliminate the gut microbiota. ABX-treated rats became easier to colonize by GMT (Figure 1). For this reason, it is important to emphasize that many studies have employed GF animals to determine the role of the gut microbiota in modulating aspects of T2DM host physiology, but this approach is, however, not suitable for our purposes. The cognitive phenotype of GF animals has obvious abnormalities, which may profoundly complicating interpretation of the effects of GMT in our model (D’Amato et al., 2020). We have therefore chosen to use ABX-mediated microbial depletion to examine the mediating role of the gut microbiota, as while removal of gut microbiota may not be as comprehensive as in GF animals, we have avoided the confounding effects caused by DACD disease. Before performing this replacement, we first confirmed that the donor ZDF rats showed T2DM phenotype and cognitive impairment at 15 weeks of age (Supplementary Figures 1, 2), and that the gut microbiota was distinguished from that of the donor LZ rats (Supplementary Figure 3). Further results were consistent with previous reports (Zhang et al., 2020), ABX treatment effectively suppressed gut microbiota activities in rats, but no changes were observed in random blood glucose (RBG), body weight (BW), or abdominal circumference (AC) (Supplementary Figure 4), indicating that the intestinal environment of the recipient rats was prepared for replacement with the new microbiota community. Interestingly, metabolic characteristics of ZDF rats showed significant changes in response to transplantation. The changes of RBG, BW and AC at different stages were observed in the ZDF rats receiving the LZ rat gut microbiota (Z-Lg group) and the ZDF rats receiving the ZDF rat gut microbiota (Z-Zg group) at week 2 after the GMT. The Z-Lg group had an decreasing trend, while the Z-Zg group had an increasing trend, compared with the ZDF model group (Z-P group) (Figures 2A–C). In addition, glycolipid metabolism phenotype of the recipient ZDF rats showed significant changes in response to transplantation, as shown in oral glucose tolerance test (OGTT)/insulin secretion test (ITT), glycosylated hemoglobin (HbA1c%), fasting serum insulin (FSI), insulin resistance index (HOMA-IR), leptin and blood biochemistry index levels (TG, TC, and LDL-C), compared with the Z-P group (Figures 2D–L). HDL-C level also was determined and difference was observed only between the recipient ZDF groups and the recipient LZ groups. However, the recipient LZ rats still maintained a relatively stable health status between LZ control group (L-P group) and LZ rats receiving LZ rat (L-Lg group) or ZDF rat (L-Zg group) after GMT (Figures 2A–L).

**FIGURE 3:**
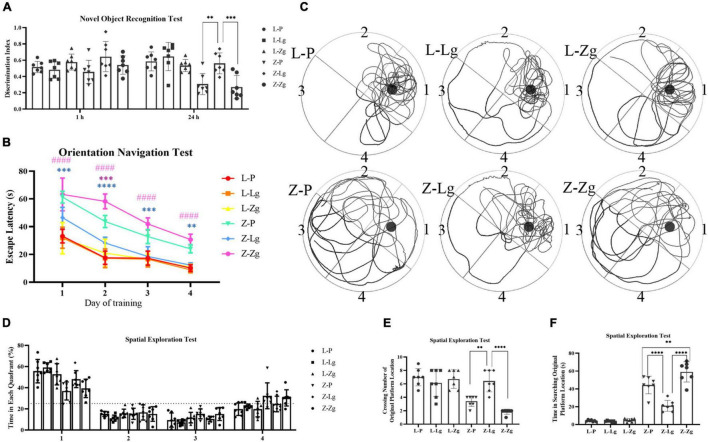
GMT changes cognitive impairment in ZDF recipient rats. **(A)** Discrimination index in the new object recognition test [*F*_(5_, _36)_ = 7.603, *P* < 0.0001]. **(B)** The escape latency during a 4 days training course in the orientation navigation test [Time: *F*_(3_, _18)_ = 171.7, *P* < 0.0001; Group: *F*_(5_, _30)_ = 115.7, *P* < 0.0001]. **(C–F)** Representative swimming path **(C)**, time spent in each quadrant (%) (dotted line indicates that the average chance of entering each quadrant is 25%) **(D)** [*F*_(5_, _30)_ = 2.082, *P* =0.0955], crossing number of the original platform location **(E)** [*F*_(5_, _36)_ = 16.82, *P* < 0.0001] and time in searching original platform location **(F)** [*F*_(5_, _36)_ = 87.94, *P* < 0.0001] in the spatial exploration test. The small circle in **(B)** represents the original platform location, but the escape platform was removed in the spatial exploration test. Data are shown as mean ± SD (*n* = 7 per group). ^**^P < 0.01, ^***^P < 0.001, ^****^P < 0.0001, compared with Z-P group; ^####^*P* < 0.0001, compared with Z-Lg group in **(C)**. ^**^P<0.01, ^****^P < in **(A,D–F)**. One-way or two-way ANOVA, followed by Tukey’s post-hoc test.

We next determined whether GMT affected cognitive function. We conducted two behavioral paradigms to assess learning and memory abilities of rats. New object recognition tests were commonly used to evaluate non-spatial memory capabilities. The results show that Z-Lg group perform better, while Z-Zg group perform worse, compared with Z-P group, as shown in the difference between the new object and a higher discrimination index (Figure 3A). Subsequently, spatial learning and memory were evaluated using Morris water maze (MWM) tests. In the orientation navigation test, rats of all groups gradually learned to find the hidden platform (escape latency) and showed a day-to-day decline in escape latency to reach the platform during the training (Figure 3B). Compared with the recipient LZ rats, the time required to find the hidden platform was significantly higher in the recipient ZDF rats, which indicated that the spatial learning and memory were impaired in the latter group. However, among the recipient ZDF groups, the Z-Lg group exhibited a shorter escape latency than the Z-P group, while the Z-Zg group showed the opposite effect (Figure 3B). On the fourth day of training, the escape latency of rats in the Z-Lg group was close to that of the recipient LZ rats (Figure 3B). Additionally, in the spatial exploration test, the Z-Lg group showed improved spatial memory performance, which was measured by crossing of the original platform location for the first time in a short time and frequent traversal of the original platform location (Figures 3C–F). Moreover, in the target quadrant, Z-Lg rats spent more time than Z-P group, although this difference was not significant (Figure 3D). The representative path of the rat during the exploration is shown in Figure 3C. In contrast, rats in the recipient LZ group showed no difference in the behavioral tests. Therefore, GMT from LZ rats and ZDF rats can change the glycolipid metabolism level and cognitive function in ZDF recipient rats, but not in LZ recipient rats.

### Gut Microbiota Transplantation Changes Leptin/Insulin Resistance and Aβ Plaque Burden

**FIGURE 4:**
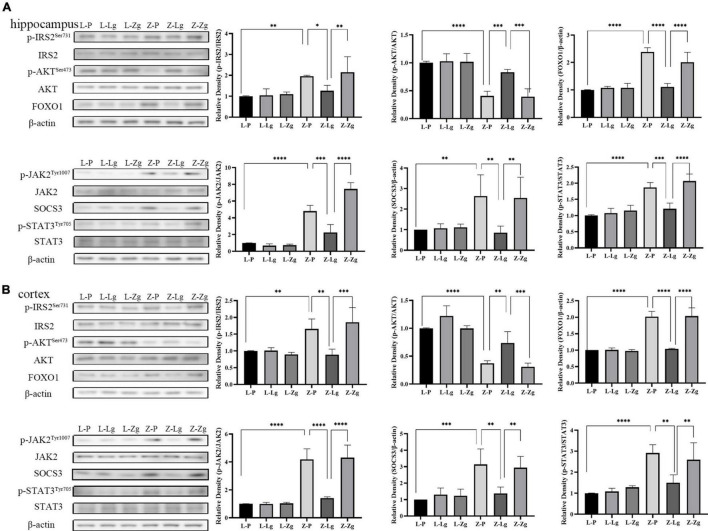
GMT changes leptin/insulin resistance in ZDF recipient rats. **(A)** Western blotting analysis of components of the insulin and leptin signaling pathway in the hippocampus regions [p-IRS2: *F*_(5_, _12)_ = 6.281, *P* = 0.0044; p-AKT: *F*_(5_, _12)_ = 23.24, *P* < 0.0001; FOXO1: *F*_(5_, _12)_ = 33.04, *P* < 0.0001; p-JAK2: *F*_(5_, _12)_ = 68.28, *P* < 0.0001; SOCS3: *F*_(5_, _12)_ = 5.399, *P* = 0.0079; p-STAT3: *F*_(5_, _12)_ = 24.59, *P* < 0.0001]. **(B)** Western blotting analysis of components of the insulin and leptin signaling pathway in the cortex regions [p-IRS2: *F*_(5_, _12)_ = 10.19, *P* = 0.0005; p-AKT: *F*_(5_, _12)_ = 28.83, *P* < 0.0001; FOXO1: *F*_(5_, _12)_ = 52.65, *P* < 0.0001; p-JAK2: *F*_(5_, _12)_ = 33.76, *P* < 0.0001; SOCS3: *F*_(5_, _12)_ = 8.807, *P* = 0.0010; p-STAT3: *F*_(5_, _12)_ = 12.40, *P* = 0.0002]. Data are shown as mean ± SD (*n* = 3 per group). *P < 0.05, ^**^P < 0.01, ^***^P < 0.001, ^****^P < 0.0001. One-way or two-way ANOVA, followed by Tukey’s *post-hoc* test.

The observation that GMT can change the behavior of rats prompted us to study the molecular mechanism of this change. The hippocampus and cortex are the major regions of the brain involved in cognition and memory (Sun et al., 2016). We examined insulin and leptin signaling pathways in the hippocampus and cortex to assess whether GMT ameliorated brain insulin resistance and leptin resistance, given that these pathways play key roles in maintaining blood glucose homeostasis and brain function. As for other parameters, we observed that the Z-Lg group showed improved insulin resistance and leptin resistance, which was achieved by increases in the protein levels of components of the IRS2-AKT-FOXO1 pathway and JAK2-SOCS3-STAT3 pathway in the hippocampus and cortex. In the recipient ZDF rats, the expression of p-IRS2, FOXO1, p-JAK2, SOCS3, and p-STAT3 increased, and the expression of p-AKT protein decreased in the Z-P group, compared with the L-P group; the Z-Lg group showed the opposite trend, compared with the Z-P group (Figures 4A,B). There were significant differences between the Z-Zg group and the Z-Lg group (Figures 4A,B). In contrast, we did not observe significant changes in these pathways in the recipient LZ rats. These effects appeared to be mediated by microbiota-induced changes in insulin resistance and leptin resistance.

**FIGURE 5:**
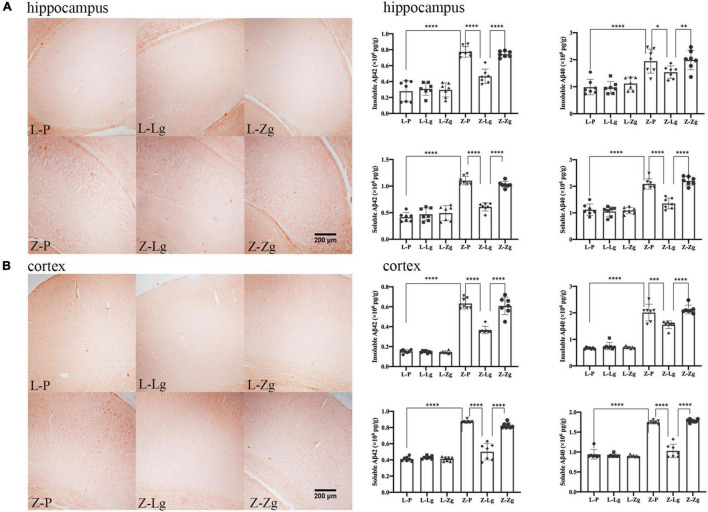
GMT changes Aβ plaque burden in ZDF recipient rats. **(A)** Representative photographs of Congo red staining (Magnification 40 ×) and levels of insoluble and soluble Aβ42 and Aβ40 in the hippocampus regions [insoluble Aβ42: *F*_(5_, _36)_ = 48.60, *P* < 0.0001; insoluble Aβ40: *F*_(5_, _36)_ = 16.32, *P* < 0.0001; soluble Aβ42: *F*_(5_, _36)_ = 66.70, *P* < 0.0001; soluble Aβ40: *F*_(5_, _36)_ = 58.05, *P* < 0.0001]. **(B)** Representative photographs of Congo red staining (Magnification 40 ×) and levels of insoluble and soluble Aβ42 and Aβ40 in the cortex regions [insoluble Aβ42: *F*_(5_, _36)_ = 177.5, *P* < 0.0001; insoluble Aβ40: *F*_(5_, _36)_ = 116.2, *P* < 0.0001; soluble Aβ42: *F*_(5_, _36)_ = 132.6, *P* < 0.0001; soluble Aβ40: *F*_(5_, _36)_ = 159.9, *P* < 0.0001]. Data are shown as mean ± SD (*n* = 3–7 per group). *P < 0.05, ^**^P < 0.01, ^***^P < 0.001, ^****^P < 0.0001. One-way or two-way ANOVA, followed by Tukey’s *post-hoc* test.

In addition, it is worth noting that amyloid plaque burden in the brain is a major pathological aspect of DACD (Bi et al., 2021). Therefore, we performed Congo red staining on the hippocampus and cortex to determine the dense amyloid plaques. In the recipient ZDF rats, the Z-Lg group showed the decreased brick-red patches in the hippocampus and cortex, although plaques still were present (Figures 5A,B). The number of plaques in the Z-Zg group was closer to that in the Z-P group (Figures 5A,B). These results also were seen in both insoluble and soluble Aβ levels as determined by enzyme-linked immunosorbent assays (ELISA) in tissues from both brain regions, with a particularly strong effect on soluble Aβ42 levels (Figures 5A,B). But in the recipient LZ rats, there was no statistical difference among all the groups. Taken together, these results indicate that GMT can affect the amyloid plaque burden of ZDF rats, which may be caused by altered brain insulin and leptin resistance.

### Gut Microbiota Transplantation Changes Gut Microbial Composition

**FIGURE 6:**
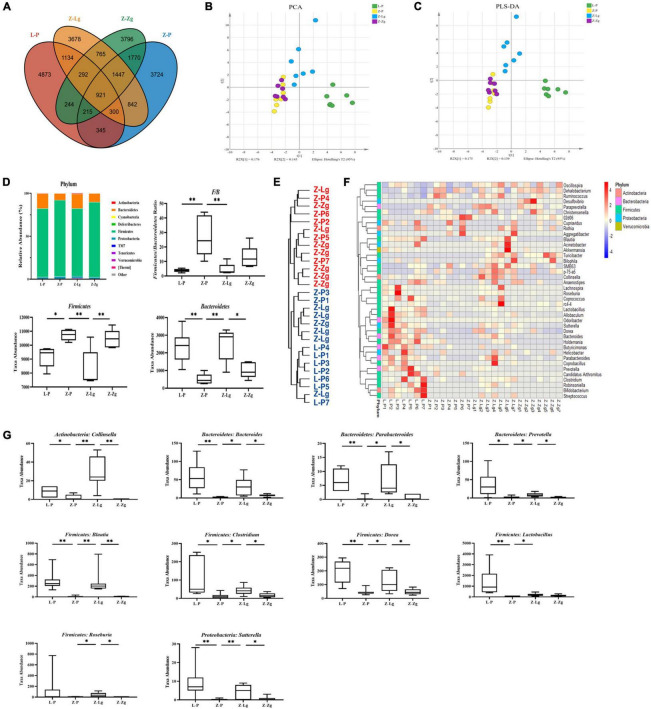
GMT changes gut microbial composition in ZDF recipient rats. **(A)** The number of OTUs that are distinct and shared across the groups by Venn diagrams. **(B,C)** Microbiota community analysis based on PCA and PLS-DA score plots. **(D)** Bacterial taxonomic composition profiling of gut microbiota at the phylum level [*F/B*: *F*_(3_, _16)_ = 8.758, *P* = 0.0011; *Firmicutes*: *F*_(3_, _16)_ = 8.744, *P* = 0.0012; *Bacteroidetes*: *F*_(3_, _17)_ = 9.512, *P* = 0.0006]. **(E)** Phylogenetic tree map of the bacterial at the genus level. **(F)** Hierarchical clustering heatmap of the top 50 differentiated taxa at the genus level. **(G)** Abundance of the key differentiated taxa in different groups [*Collinsella*: *F*_(3_, _24)_ = 14.75, *P* < 0.0001; *Bacteroides*: *F*_(3_, _22)_ = 6.924, *P* = 0.0019; *Parabacteroides*: *F*_(3_, _23)_ = 7.297, *P* = 0.0014; *Prevotella*: *F*_(3_, _21)_ = 5.323, *P* = 0.0069; *Blautia*: *F*_(3_, _24)_ = 8.530, *P* = 0.0005; *Clostridium*: *F*_(3_, _22)_ = 5.186, *P* = 0.0073; *Dorea*: *F*_(3_, _23)_ = 9.372, *P* = 0.0003; *Lactobacillus*: *F*_(3_, _21)_ = 5.360, *P* = 0.0067; *Roseburia*: *F*_(3_, _24)_ = 1.438, *P* = 0.2564; *Sutterella*: *F*_(3_, _24)_ = 5.924, *P* = 0.0036]. Data are shown as mean ± SD (*n* = 5–7 per group). *P<0.05, ^**^P<0.01. One-way or two-way ANOVA, followed by Tukey’s *post-hoc* test. OTUs, operational taxonomic units; PCA, principal component analysis; PLS-DA, partial least squares discriminant analysis.

Next, 16S rRNA gene-based sequencing was used to analyze the differences in microbial composition profiles of intestinal contents samples from 42 recipient rats and 24 donor rats. The results showed that the recipient L-P group and the donor L group were similar in the main components of PC2, and the GMT did not change the intestinal microbial composition of the recipient L-Lg group and L-Zg group; the Z-P group is similar to the donor Z group in terms of the main components of PC2, the intestinal microbial composition of the Z-Lg group transplanted with healthy gut microbiota is closer to that of the donor L group, and the intestinal microbial composition of the Z-Zg group is closer to the donor Z group, as the same way (Supplementary Figure 5). Therefore, we investigated variations in the structural diversity of the 4 groups of rats (L-P group, Z-P group, Z-Lg group, Z-Zg group) with phenotypic changes, focusing on microbiota believed to be involved in the pathogenesis of DACD. Although the total number of operational taxonomic units (OTUs) initially remained at a consistent level, differences in community structure were observed among different experimental groups (Figure 6A). It is worth noting that the community diversity of the Z-Lg group has been significantly improved and the community structure of the species tends to be normal after GMT, while the community structure of the Z-Zg group tends to be more toward the Z-P group (Figures 6B,C), suggesting that transplanting the healthy gut microbiota can improve the structure disorder of ZDF rats. The relative abundance of the discriminatory taxa were assessed by microbial taxonomic analysis. At the phylum level, the main taxonomic units were *Firmicutes* and *Bacteroidetes*, which account for more than 96% of all bacterial species (Figure 6D). The ratio of *Firmicutes* to *Bacteroidetes* (*F/B*), which is affected by the increased abundance of *Firmicutes* and/or decreased in *Bacteroidetes*, has been described as a characteristic of intestinal ecological disorders (Cani, 2019). Notably, this parameter was improved in the Z-Lg group, compared with Z-P group (Figure 6D). The heatmap showed the differentiated taxa with horizontal abundance, and the phylogenetic tree map showed the differences among the groups. The results showed that the microbial community composition of the L-P group and the Z-P group was significantly different. Among them, Z-Lg group was toward to the L-P group, while the Z-Zg group was toward to the Z-P group (Figures 6E,F). At the genus level, we detected 10 genera that exhibited significant differences in taxa abundances (Figure 6G), of which 1 specie (*Collinsella*) belonged to *Actinobacteria*, 3 species (*Bacteroides*, *Parabacteroides*, *Prevotella*) belonged to *Bacteroidetes*, 5 species (*Blautia*, *Clostridium*, *Dorea*, *Lactobacillus*, *Roseburia*) belong to *Firmicutes*, and 1 species (*Sutterella*) belongs to *Proteobacteria*, which may represent a core group of bacteria that contribute to the phenotypic differences among the recipient animals. Our results indicated that GMT has the potential to alter the gut microbiota composition of recipient ZDF rats, contributing to the maintenance of physical health and metabolic stability, although further research will be needed to confirm this hypothesis.

### Gut Microbiota Transplantation Changes Metabolite Composition

**FIGURE 7:**
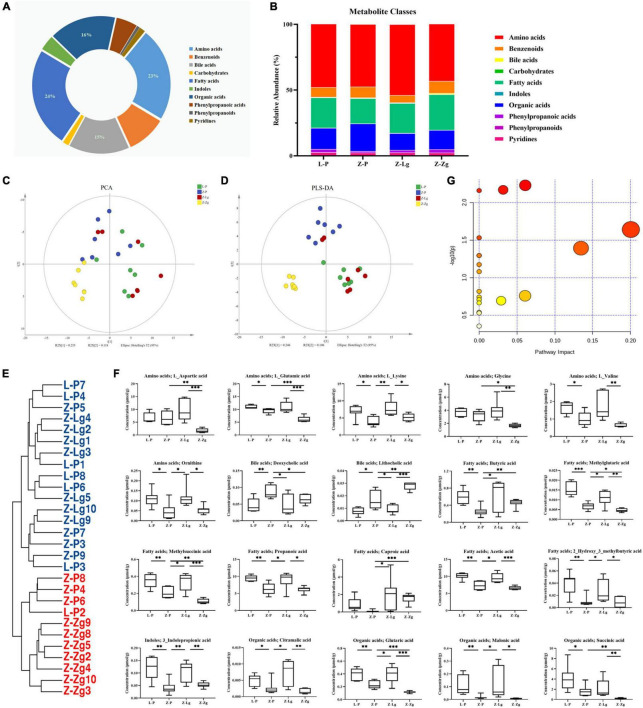
GMT changes metabolite composition of gut microbiota in ZDF recipient rats. **(A,B)** Composition of metabolite classes analysis. **(C,D)** Global metabolite analysis based on PCA and PLS-DA score plots. **(E)** Phylogenetic tree map of the metabolites. **(F)** Differential metabolites [L_aspartic acid: *F*_(3_, _20)_ = 9.366, *P* = 0.0005; L_glutamic acid: *F*_(3_, _18)_ = 16.26, *P* < 0.0001; L_lysine: *F*_(3_, _22)_ = 5.884, *P* = 0.0042; glycine: *F*_(3_, _20)_ = 8.070, *P* = 0.0010; L_valine: *F*_(3_, _20)_ = 6.989, *P* = 0.0021; ornithine: *F*_(3_, _24)_ = 5.983, *P* = 0.0034; deoxycholic acid: *F*_(3_, _24)_ = 5.075, *P* = 0.0073; lithocholic acid: *F*_(3_, _22)_ = 28.50, *P* < 0.0001; butyric acid: *F*_(3_, _24)_ = 4.146, *P* = 0.0168; methylglutaric acid: *F*_(3_, _24)_ = 20.94, *P* < 0.0001; methylsuccinic acid: *F*_(3_, _22)_ = 16.14, *P* < 0.0001; propionic acid: *F*_(3_, _24)_ = 8.244, *P* = 0.0006; caproic acid: *F*_(3_, _21)_ = 3.217, *P* = 0.0436; acetic acid: *F*_(3_, _22)_ = 16.67, *P* < 0.0001; 2-hydroxy-3-methylbutane acid: *F*_(3_, _24)_ = 7.715, *P* = 0.0009; 3-indolepropionic acid: *F*_(3_, _24)_ = 10.24, *P* = 0.0002; citramalic acid: *F*_(3_, _24)_ = 7.506, *P* = 0.0010; glutaric acid: *F*_(3_, _22)_ = 17.92, *P* < 0.0001; malonic acid: *F*_(3_, _24)_ = 5.205, *P* = 0.0065; succinic acid: *F*_(3_, _23)_ = 6.524, *P* = 0.0024]. **(G)** Diagram of the metabolic enrichment pathways. Data are shown as mean ± SD (*n* = 5–7 per group). *P < 0.05, ^**^P<0.01, ^***^P < 0.001. One-way or two-way ANOVA, followed by Tukey’s *post-hoc* test. PCA, principal component analysis; PLS-DA, partial least squares discriminant analysis.

GMT treatment has a large impact on the gut microbiota, raising our interest in investigating whether and how GMT affected microbial metabolites in the circulation of the recipient animals. We subsequently used the ultra-performance liquid chromatography coupled to tandem mass spectrometry (UPLC-MS/MS) protocol to perform targeted metabolomics analysis on the intestinal contents samples, and a total of 136 metabolites in 28 samples were ultimately identified and quantified, including amino acids, benzoates, bile acids, carbohydrates, fatty acids, indole, organic acids, phenylpropionic acid, phenylpropane and pyridines (Figures 7A,B). Among them, amino acids, fatty acids and organic acids were the major metabolite types, accounting for approximately 87% of all metabolites (Figures 7A,B). In order to evaluate the difference among the groups, we used discriminant analysis of the metabolic structure of each group of samples. A principal component analysis (PCA) and partial least squares discriminant analysis (PLS-DA) model showed clear separation among the groups, despite rearing in the same environment (Figures 7C,D). The metabolic profile of the Z-Lg group was toward to the L-P group, while the Z-Zg group was toward to the Z-P group. This was confirmed in the phylogenetic tree diagram (Figure 7E). Pairwise orthogonal partial least squares discriminant analysis (OPLS-DA) models were constructed and full-spectrum information for all samples was subjected to 200 rounds of model replacement test analysis to ensure that the model was predictive and reliable (Supplementary Figure 6). VIP > 1 and *P* < 0.05 were used to identify differential metabolites in intestinal content samples. The results showed that GMT changed the concentration of 20 metabolites, involving amino acid metabolism (L_aspartic acid, L_glutamic acid, L_lysine, glycine, L_valine, ornithine), bile acid metabolism (deoxycholic acid, lithocholic acid), fatty acid metabolism (butyric acid, methylglutaric acid, methylsuccinic acid, propionic acid, caproic acid, acetic acid, 2-hydroxy-3-methylbutane acid), indole metabolism (3-indolepropionic acid), and organic acid metabolism (citramalic acid, glutaric acid, malonic acid, succinic acid) (Figure 7F). The potential differential metabolites and their detailed information are listed in Table 1. Based on the results of the potential differential metabolites, the web-based MetaboAnalyst 4.0 system was used for relevant pathway analysis. The main change pathways detected were concentrated in 14 disturbed metabolic pathways (*P* < 0.1) (Figure 7G and Table 2). There is no doubt that gut microbiota transplantation will affect the metabolic structure of the recipients, and the most significant changes were fatty acid metabolism and amino acid metabolism. These metabolites presumably participate (directly or indirectly) in the production and metabolic reactions of proteins, lipids, and carbohydrates and provide energy for the recipient body.

**TABLE 1:** Potential differential metabolites.

Class	Metabolites	HMDBID	KEGG ID	VIP	FC (L-P/Z-P)	FC (Z-P/Z-Lg)	FC (Z-P/Z-Zg)	FC (Z-Lg/Z-Zg)
Amino acids	L-Aspartic acid	HMDB0000191	C00049	1.34	0.02	0.56	2.19	2.63
	L-Glutamic acid	HMDB0000148	C00025	1.26	1.13	0.47	2.54	2.42
	L-Lysine	HMDB0000182	C00047	1.24	1.51	1.81	0.6	1.14
	Glycine	HMDB0000123	C00037	1.21	0.86	0.78	1.2	2.23
	L-Valine	HMDB0000883	C00183	1.07	1.2	0.75	0.95	1.7
	Ornithine	HMDB0000214	C00077	1.04	1.51	1.39	0.13	1.47
Bile acids	Deoxycholic acid	HMDB0000626	C04483	1.36	1.88	1.45	1.33	0.37
	Lithocholic acid	HMDB0000761	C03990	1.2	1.29	1.15	1.87	5.25
Fatty acids	Butyric acid	HMDB0000039	C00246	1.37	2.24	1.18	1.72	0.42
	Methylglutaric acid	HMDB0000752	NA	1.32	3.13	1.1	1.43	2.16
	Methylsuccinic acid	HMDB0001844	C08645	1.27	1.74	1.17	1.87	2.87
	Propanoic acid	HMDB0000237	C00163	1.24	2.3	1.07	0.11	1.45
	Caproic acid	HMDB0000535	C01585	1.2	0.93	1.04	3.34	0.13
	Acetic acid	HMDB0000042	C00033	1.2	2.31	1.36	0.48	2.73
	2-Hydroxy-3-methylbutyric acid	HMDB0000407	NA	1.09	2.05	1.09	0.07	1.19
Indoles	3-Indolepropionic acid	HMDB0002302	NA	1.35	2.16	1.95	0.34	1.91
Organic acids	Citramalic acid	HMDB0000426	C00815	1.3	1.11	1.32	0.47	1.95
	Glutaric acid	HMDB0000661	C00489	1.29	2.04	1.29	2.37	2.87
	Malonic acid	HMDB0000691	C04025	1.11	1.7	1.08	0.8	1.23
	Succinic acid	HMDB0000254	C00042	1	1.02	0.29	1.97	1.62

**TABLE 2:** Metabolite pathway.

No.	Pathway	Total	Hits	Raw p	Log(p)	Holm p	FDR	Impact
1	Aminoacyl-tRNA biosynthesis	48	5	0.00	3.91	0.01	0.01	0.00
2	Arginine biosynthesis	14	3	0.00	3.40	0.03	0.01	0.18
3	Butanoate metabolism	15	3	0.00	3.30	0.04	0.01	0.00
4	Glutathione metabolism	28	3	0.00	2.49	0.27	0.05	0.11
5	Alanine, aspartate and glutamate metabolism	28	3	0.00	2.49	0.27	0.05	0.42
6	Glyoxylate and dicarboxylate metabolism	32	3	0.00	2.32	0.38	0.07	0.11
7	Histidine metabolism	16	2	0.01	1.88	1.00	0.16	0.00
8	Pantothenate and CoA biosynthesis	19	2	0.02	1.74	1.00	0.19	0.00
9	Propanoate metabolism	23	2	0.03	1.58	1.00	0.25	0.00
10	Porphyrin and chlorophyll metabolism	30	2	0.04	1.36	1.00	0.36	0.00
11	Nitrogen metabolism	6	1	0.07	1.18	1.00	0.43	0.00
12	D-Glutamine and D-glutamate metabolism	6	1	0.07	1.18	1.00	0.43	0.50
13	Arginine and proline metabolism	38	2	0.07	1.18	1.00	0.43	0.20
14	Valine, leucine and isoleucine biosynthesis	8	1	0.09	1.06	1.00	0.52	0.00

*Hits represents the matched number of metabolites in one pathway. Raw P represents the original P-value calculated from the enrichment analysis. Holm P represents the P-value further adjusted using Holm-Bonferroni method. FDR P represents the P-value adjusted using false discovery rate.*

### Diabetes-Associated Cognitive Decline Bioinformatics Analysis and Correlation Network Construction Based on the Mechanism of Microbiota-Gut-Brain Axis

**FIGURE 8:**
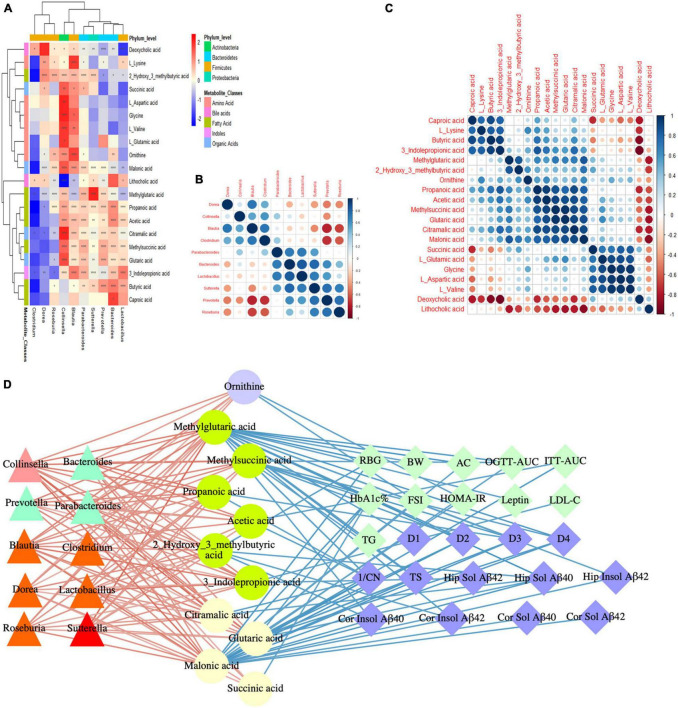
DACD bioinformatics analysis and correlation network construction. **(A)** Correlation between differential genera and metabolites. **(B)** Spearman correlation analysis of differential genera. **(C)** Spearman correlation analysis of differential metabolites. **(D)** Correlation among bacterial genera (triangle), intestinal metabolites (circle), and DACD phenotypes (diamond). The degree of correlation is shown by the gradient change of red (positive correlation) and blue (negative correlation). *P < 0.05, ^**^P < 0.01, ^****^P < 0.0001. RBG, random blood glucose; BW, body weight; AC, abdominal circumference; OGTT-AUC, area under the curve of the oral glucose tolerance test; ITT-AUC, area under the curve of the insulin secretion test; HbA1c%, glycosylated hemoglobin; FSI, fasting serum insulin; HOMA-IR, homeostasis model of assessment for insulin resistance; D1-D4, escape latency during a 4 days training course in the orientation navigation test of Morris water maze test; 1/CN, the reciprocal of crossing number of the original platform location in the spatial exploration test of Morris water maze test; TS, time in searching original platform location in the spatial exploration test of Morris water maze test; Hip, hippocampus; Cor, cortex.

To study the effects of the gut microbiota and its metabolites on the host phenotype during the development of DACD, we performed spearman correlation analysis. Based on the hierarchical clustering heatmap of the correlation between different bacterial genera and metabolites, we found that *Colinsella* and *Blautia* were positively correlated with almost all different metabolites; *Parabacteroides*, *Bacteroides*, *Lactobacillus*, *Sutterella*, and *Prevotella* were positively correlated with fatty acids and organic acids (Figure 8A), indicating that there was a potential correlation between bacterial genera and metabolites, without considering the influence of the relative abundance of the different groups. These results were reflected in the cluster heatmap of differential genera and differential metabolites. The clustering heatmap of the different genera showed: *Colinsella* and *Blautia*; *Parabacteroides*, *Bacteroides*, *Lactobacillus*, *Sutterella*, and *Prevotella* clustered into two related clusters, which may have related biological functions (Figure 8B). The clustering heatmap of the differential metabolites showed: caproic acid, L_lysine, butyric acid, and 3_indolepropionic acid; methylglutaric acid, and 2_hydroxy_3_methylbutyric acid; propanoic acid, acetic acid, methylsuccinic acid, glutaric acid, citramalic acid, and malonic acid; succinic acid, L_glutamic acid, glycine, L_aspartic acid, and L_valine clustered into four related clusters, respectively, exerting the biological effects of microbiota-derived metabolites (Figure 8C). Among them, 10 species of genera, such as *Colinsella*, *Parabacteroides*, *Blautia*, *Lactobacillus*, *Sutterella*, can regulate 11 kinds of metabolites, such as ornithine, propanoic acid, acetic acid, citramalic acid, were significant negative with host phenotype of DACD (Figure 8D). Thus, our data demonstrated that GMT reshapes the gut microbiota structure and changes the host metabolism. The structure of the gut microbiota showed significant taxonomic perturbations, which may be the main reason for the significant changes in metabolomics characteristics. These metabolic dysfunctions affect the state of glycolipid metabolism, presumably contributing to diminished cognitive function.

## Discussion

Although the composition of the gut microbiota is affected by the host genetic factors and environmental risk factors, the gut microbiota can be reshaped throughout life, depending on many factors, including diet, antibiotics, and gastrointestinal diseases (Fujisaka et al., 2018; Visconti et al., 2019). In recent years, continuing research on the gut microbiota has shown that changes in the gut microbiota can positively impact T2DM and DACD (Visconti et al., 2019; Liu et al., 2020). Metabolites are also important signals that reflect changes in the host’s microenvironment and help understand the pathogenesis of diseases. However, whether GMT can alleviate DACD-related metabolic disorders and cognitive deficits, and the role played by gut microbiota in this process, remain unclear. These challenges highlight the urgent need to investigate the mechanisms underlying how GMT counteracts DACD. The present study demonstrated, for the first time (to our knowledge), that GMT regulated cognitive functions of the central nervous system through the microbiota-gut-brain axis mechanism during the development of T2DM to DACD. These observations prompted us to investigate its underlying mechanisms and whether the observed cognitive and behavioral changes are related to the activation of insulin and leptin signaling pathways. Our results imply that complicated signaling cascades via the microbiota-gut-brain axis are influential, but are not strict determinants of the fate of particular members of the gut microbiota. It is also point to the importance of microbial ecology, microbiota-derived metabolites, or microbial function capacity (rather than the importance of a single bacterial taxon) for the pathogenesis of DACD (Sherwin et al., 2016).

The use of GMT in human medical treatment is gaining popularity, although this approach is not novel (Krajicek et al., 2019). Some 1,700 years ago, Ge Hong, a traditional Chinese medical doctor, documented the treatment of patients with food poisoning and severe diarrhea via oral administration of human fecal suspensions (Zhang et al., 2012). In moving from the clinic to the laboratory, GMT has opened up possibilities for transferring various metabolic properties and behavioral phenotypes by altering the composition of the gut microbiota, providing prospects for research and drug treatment (Ooijevaar et al., 2019; Mangioni et al., 2021; Olekhnovich et al., 2021). Here, we first demonstrated that it is possible to reshape the microbiota composition and restore healthy conditions by transplanting the gut mictobiota from donor LZ rat into the intestinal tract of the recipient ZDF rat. GMT significantly changed the characteristics of glycolipid metabolism in ZDF rats, and the level of these basic metabolisms was confirmed to be closely related to diabetes-related cognitive decline.

Our previous studies have shown that transplantation of normal gut microbiota can improve insulin and leptin signaling in the liver tissue of ZDF rats (Zhang et al., 2020). Current research indicates that similar changes occur in the brain. Indeed, insulin plays an important role in brain physiology and cognition (Kullmann et al., 2016, 2020). Insulin binds to the insulin receptor (IR), leading to the activation of insulin receptor substrate proteins (IRS) and subsequent phosphatidylinositol 3-kinase (PI3K) -mediated phosphorylation and activation of protein kinase B (AKT), which contributes to central control of the body’s energy homeostasis while also directly influencing learning and memory (Akhtar and Sah, 2020). In addition, it has been reported that leptin is secreted primarily by adipose tissue and plays a key role in maintaining energy balance and body weight (Smith et al., 2019). Leptin relays information regarding peripheral energy stores in the central nervous system, especially to the hippocampus and cortex regions, thereby regulating synaptic plasticity, learning, and memory (Mejido et al., 2020). Central leptin resistance may play a role in the pathophysiology of DACD, in which the Janus kinase (JAK) (Jakati et al., 2021)/signal transducer and activator of transcription (STAT) pathway contributes to chronic metabolic diseases (Dodington et al., 2018; Moshapa et al., 2019). Leptin binding to LepR activates JAK2, which in turn phosphorylates STAT3 (Griveau et al., 2020). STAT3, which is recruited by leptin, is a component of a major signaling pathway; due to its responsivity, STAT3 phosphorylation has become an alternative marker for the identification of leptin-responsive cells and leptin sensitivity. We found that GMT stimulated IRS2/AKT-mediated insulin signaling and JAK2/STAT3-mediated leptin signaling, providing an essential link between basal metabolism and cognitive function in the brain. At the same time, GMT (as expected) changed the accumulation of toxic Aβ in the brains and cognitive deficits of ZDF rats, especially soluble Aβ42 levels in the hippocampus and cortex, further demonstrating the effects of GMT for DACD. The accumulation of Aβ deposition in the brain is the main pathological manifestation for the decline of cognitive function in neurodegenerative diseases such as DACD and AD, which is mainly composed of β-secretase and γ-secretase through sequential cleavage of APP, resulting in different Aβ42 and Aβ40 products, respectively. While Aβ40 is the predominant form produced, Aβ42 is the predominant pathogenic form of cognitive decline. In addition, Aβ in the brain usually exists in both insoluble (insoluble fibers, deposits, and dense plaques) and soluble (monomers, dimers, trimers, oligomers, and partially soluble fibers) forms due to its aggregation-precipitation properties. Among them, insoluble Aβ fibers have neurotoxicity, which changes neuronal ion channels, causing disturbances in the neuron internal environment, accelerating the phosphorylation of Tau protein, and leading to neurofibrillary tangles; while soluble Aβ is more toxic than its fibrous deposits formed later, which affects synaptic function and may be the basis for early cognitive deficits. Recent studies have also confirmed that brain soluble Aβ42 and Aβ42/Aβ40 ratio are closely related to cognitive pathology in AD patients, rather than total Aβ levels (Kwak et al., 2020; Sturchio et al., 2021). Our findings also explain why preclinical and clinical studies of therapies focused solely on altering the production and clearance of Aβ have suggested that such treatments are insufficient to treat cognitive impairment (Yu et al., 2019; Kim et al., 2020). We infer that the gut microbiota is essential for the pathogenesis of DACD (Desbonnet et al., 2015). In summary, our results indicate that GMT can alter cognitive deficits and Aβ deposition in ZDF rats, effects that may be achieved through improved insulin resistance and leptin resistance (Chen et al., 2019). Ultimately, the effect of the gut microbiota on the key functions of the central nervous system is enhanced.

There is a social network among the core gut bacteria of healthy hosts, which maintains the balance of the host’s gut microenvironment. Recent studies have confirmed that the healthy adults gut microbiota is noted for its ecological stability, consisting of a relatively constant “core” which is resistant to changes in its composition, restore to its initial composition, or is able to recover its initial function despite compositional changes when facing disturbances due to external pressure (Moya and Ferrer, 2016). This stability is considered essential to host health as it allows the gut microbiota to respond to disturbances and failure to do so results in an irreversible dysbiotic state. It has been reported in inflammatory diseases of the gastrointestinal tract (Halfvarson et al., 2017). Similarly, our research found that GMT has no effect on healthy LZ rats, which may be because healthy individuals can respond to the transplanted gut microbiota. However, the phenotypic changes observed in ZDF rats indicate that the intestinal microbial changes lack resilience to the disturbances of its stable state, which may lead to altered the microbiota-gut-brain axis during the development of DACD disease, resulting in cognition defect. In the current study, we have found that GMT alters the abundance of certain bacteria in DACD disease-susceptible hosts, contributing to the health of ZDF rats. *Firmicutes* have been proven to be the most dominant type of bacteria in the intestines of humans and rodents. Among them, *Blautia*, *Clostridium*, *Dorea*, *Lactobacillus*, *Roseburia* are the main genus producing short-chain fatty acids (SCFA), which provide the host with energy. In addition, *Blautia* has also been shown to be closely related to AD pathology and can affect the central and peripheral Aβ levels of APP/PS1 mice. As our research shows, this may serve as a potential gut microbiota marker for neurological diseases. *Clostridium*, *Dorea* and *Roseburia* were the genera predominantly found in the gut microbiota of healthy individuals, which were negatively related to most of the characteristics of obesity, brain metabolism disorders and cognitive dysfunction (Chen et al., 2020; Sun M. et al., 2020), and were closely related to the production of butyrate (Ren et al., 2020; Xie et al., 2020). *Lactobacillus* has been shown to be a positive predictor of cognitive function, which can affect the behavior of rodents, including reducing anxiety-related symptoms and improving memory. This may be due to its ability to acidify the intestinal environment, produce essential amino acids, and protect the intestinal barrier. Related to reducing the entry of endotoxins into the blood. In addition, *Bacteroides*, *Parabacteroides*, and *Prevotella* are the main and core genera of the *Bacteroidetes* phylum, which are closely related to the improvement of cognitive ability (Lippert et al., 2017; Chen et al., 2020; Dahl et al., 2020; Ren et al., 2020; Shao et al., 2020; Sun Z. Z. et al., 2020; Xie et al., 2020). The reason may also be mainly mediated by intestinal SCFA, but further proof is needed. The core microbial ecology can maintain environmental conditions sufficient to inhibit the growth of pathogenic species/pathogens and support host health, just as the interaction among murine gut microbiota has been studied in GK rats (Draper et al., 2018).

GMT led to alterations in microbial metabolites, which might further explain the remote effect of GMT on the cognitive impairment of DACD. We detected changes in the concentration of various metabolites after GMT, and different metabolites are mainly fatty acids, amino acids and organic acids. Evidence had shown that abnormal lipid metabolism has an important impact on neurological diseases, especially cognitive function (Rojas et al., 2019). Interestingly, we found that GMT can regulate the levels of multiple fatty acids, and we herein identified that the levels of SCFAs (butyric acid, propionic acid, caproic acid, caproic acid) were significantly positively correlated with the abundance of *Colinsella*, *Blautia*, *Parabacteroides*, *Bacteroides*, *Lactobacillus*, *Sutterella*, and *Prevotella*. SCFA is mainly produced from the diet by intestinal microorganisms (i.e., intestinal symbiotic bacteria and/or probiotic bacteria). Acetic acid metabolism is the main source of host energy; propionic acid is catabolized in the liver after being absorbed by the blood, and participates in the process of reverse conversion of pyruvate to glucose, while inhibiting the synthesis of fat; butyric acid has been proven to prevent obesity and insulin resistance. These are all factors of cognitive dysfunction. As far as we know, there is very little information available regarding the role of amino acid metabolism in diabetic cognitive dysfunction, but amino acids never be ignored in the research field of neurological diseases (Kawase et al., 2017). The nervous system mainly used neurotransmitters to transmit information, and amino acid-mediated neurotransmitters were widely distributed in the nervous system. Therefore, amino acids are essential for nerve conduction, receptor function, learning and memory. Our research found that GMT regulates the level of amino acid metabolism. For example, glutamic acid and aspartic acid have been identified as two excitatory neurotransmitters related to cognitive impairment (Mazzoli and Pessione, 2016), and the changes in metabolic levels have been confirmed in type 2 diabetes models in db/db mice (Zheng et al., 2017b,c) and STZ-induced rats (Zheng et al., 2017a). Glycine is an inhibitory neurotransmitter that plays an important role in the inhibitory and excitatory synapses of the central nervous system (Eulenburg et al., 2005). Glycine transporter may be novel therapeutic targets in neurological diseases. Valine is a branched-chain amino acid that can quickly provide amino groups to synthesize glutamic acid. It has been reported to decrease in cognitive impairment and indirectly affect the transmission of excitatory neurotransmitters. This may also be a possible explanation for the decline of cognitive function in diabetes. The vital role of energy metabolism is unquestionable for maintaining the normal life activities in mammals. In this study, we found that GMT changes the energy metabolism response of ZDF rats, as indicated by the significant changes in TCA cycle intermediates, citric acid, glutaric acid, malonic acid, and succinic acid. It has been well known that energy metabolism plays a key role in cognitive function (Kapogiannis and Mattson, 2011). Therefore, we speculate that the energy metabolism disorder improved by GMT may be the reason for the improved cognitive function of diabetes, which is consistent with previous research (Zheng et al., 2017c). In addition, another fascinating discovery is that changes in energy metabolism are inseparable from changes in gut microbiota, including citric acid, glutaric acid, and malonic acid (Cani et al., 2019). The changes of co-metabolites in the body reflect changes in the gut microbiota (Sieow et al., 2019). It also can be said that the metabolic phenotype is closely related to the gut microbiota. Various genera and metabolites that are closely related may be used as biomarkers, facilitating exploration of the involvement of gut microbiota in host pathogenesis.

Overall, the research described in the present work suggests a novel link between gut microbiota and cognitive function, a link that is mediated via changes in microbial metabolite levels following treatment by GMT in recipient ZDF rats. GMT re-structures the gut microbiota and alters the microbial metabolite profile, improving basal metabolism, counteracting insulin resistance and leptin resistance, reducing Aβ deposition in the brain, and alleviating cognitive and spatial memory disorders. The impact of GMT on changes in microbiota and metabolites will need to be further evaluated in clinical trials, and additional analysis will be needed to distinguish functionally critical taxa from the general microbiota. Screening of probiotic candidates and GMT components is expected to permit translation into a novel ecological approach for managing metabolic and neurological diseases.

## Data Availability Statement

The datasets presented in this study can be found in online repositories. The names of the repository/repositories and accession number(s) can be found in the article/Supplementary Material.

## Ethics Statement

All animal procedures were performed at Nanjing University of Chinese Medicine (Nanjing, China) in accordance with the National Institutes of Health Guide for the Care and Use of Laboratory Animals and a study-specific animal protocol that was approved by the Animal Ethics Committee of Nanjing University of Chinese Medicine (Approval No. 201812A009).

## Author Contributions

LBZ and XL conceived the idea and designed the study. TB, RF, TZ, WR, and TH performed the experiments, obtained the samples, and acquired the data. TB, LJZ, and WZ performed the microbiomics and metabolomics data analysis. TB conducted molecular biology experiments and wrote the manuscript. LBZ directed the project and involved in modifying the manuscript. All authors had approved the final manuscript for submission.

## Conflict of Interest

The authors declare that the research was conducted in the absence of any commercial or financial relationships that could be construed as a potential conflict of interest.

## Publisher’s Note

All claims expressed in this article are solely those of the authors and do not necessarily represent those of their affiliated organizations, or those of the publisher, the editors and the reviewers. Any product that may be evaluated in this article, or claim that may be made by its manufacturer, is not guaranteed or endorsed by the publisher.

## Supplementary Material

Supplementary Figure 1 Basal metabolism phenotype of the donor rats. **(A)** Random blood glucose (RBG; *t* = 16.56, df = 22, *P* < 0.0001). **(B)** Body weight (BW; *t* = 9.203, df = 22, *P* < 0.0001). **(C)** Abdominal circumference (AC; *t* = 8.333, df = 22, *P* < 0.0001). **(D)** Oral glucose tolerance test [OGTT; Time: *F*_(4, 88)_ = 133.1, *P* < 0.0001; Group: *F*_(1, 22)_ = 484.1, *P* < 0.0001]. **(E)** Insulin secretion test [ITT; Time: *F*_(3.154, 69.38)_ = 91.55, *P* < 0.0001; Group: *F*_(1, 22)_ = 308.6, *P* < 0.0001]. **(F)** Area under the curve of the oral glucose tolerance test (OGTT-AUC; *t* = 22.62, df = 22, *P* < 0.0001). **(G)** Area under the curve of the insulin secretion test (ITT-AUC; *t* = 14.28, df = 22, *P* < 0.0001). **(H)** Glycosylated hemoglobin (HbA1c%; *t* = 12.38, df = 22, *P* < 0.0001). **(I)** Fasting serum insulin (FSI; *t* = 4.581, df = 22, *P* = 0.0001). **(J)** Homeostasis model of assessment for insulin resistance index (HOMA-IR; *t* = 6.246, df = 22, *P* < 0.0001). **(K)** Leptin (*t* = 11.05, df = 22, *P* < 0.0001). **(L)** Triglyceride (TG; *t* = 6.760, df = 22, *P* < 0.0001), total cholesterol (TC; *t* = 6.962, df = 22, *P* < 0.0001), low-density lipoprotein cholesterol (LDL-C; *t* = 9.992, df = 22, *P* < 0.0001) and high-density lipid cholesterol (HDL-C; *t* = 3.918, df = 22, *P* = 0.0007). Data are shown as mean ± SD (*n* = 12 per group). ^***^*P*<0.001, ^****^*P*<0.0001. Two-tailed unpaired Student’s *t*-test.

Supplementary Figure 2 Cognitive function of the donor rats. **(A)** Discrimination index in the new object recognition test (*t* = 1.083, df = 14, *P* = 0.2973). **(B)** The escape latency during a 4 days training course in the orientation navigation test [Time: *F*_(2.348, 51.65)_ = 38.69, *P* < 0.0001; Group: *F*_(1, 22)_ = 87.86, *P* < 0.0001]. **(C–F)** Representative swimming path **(C)**, time spent in each quadrant (%) (dotted line indicates that the average chance of entering each quadrant is 25%) **(D)** (*t* = 4.127, df = 22, *P* = 0.0004), crossing number of the original platform location **(E)** (*t* = 5.250, df = 22, *P* < 0.0001) and time in searching original platform location **(F)** (*t* = 3.599, df = 22, *P* = 0.016) in the spatial exploration test. The small circle in Figure 2C represents the original platform location, but the escape platform was removed in the spatial exploration test. Data are shown as mean ± SD (*n* = 8–12 per group). ^**^*P* < 0.01, ^****^*P* < 0.0001. Two-tailed unpaired Student’s *t*-test.

Supplementary Figure 3 Gut microbiota structure of the donor rats. **(A)** The number of OTUs that are distinct and shared across the groups by Venn diagrams. **(B,C)** Microbiota community analysis based on PCA and PLS-DA score plots. **(D)** Bacterial taxonomic composition profiling of gut microbiota at the phylum level (*F/B*: *t* = 2.444, df = 22, *P* = 0.0230; *Firmicutes*: *t* = 2.311, df = 20, *P* = 0.0316; *Bacteroidetes*: *t* = 2.483, df = 22, *P* = 0.0211). **(E)** Phylogenetic tree map of the bacterial at the genus level. **(F)** Hierarchical clustering heatmap of the top 50 differentiated taxa at the genus level. Data are shown as mean ± SD (*n* = 12 per group). **P*<0.05. Two-tailed unpaired Student’s *t*-test. OTUs, operational taxonomic units; PCA, principal component analysis; PLS-DA, partial least squares discriminant analysis.

Supplementary Figure 4 Basal phenotype and gut microbiota composition before and after GMT in ZDF recipient rats. **(A)** Random blood glucose [RBG; Time: *F*_(1, 6)_ = 9.809, *P* = 0.0203; Group: *F*_(5, 30)_ = 207.6, *P* < 0.0001]. **(B)** Body weight [BW; Time: *F*_(1, 6)_ = 285.0, *P* < 0.0001; Group: *F*_(5, 30)_ = 40.09, *P* < 0.0001]. **(C)** Abdominal circumference (AC; Time: *F*_(1, 6)_ = 14.23, *P* = 0.0093; Group: *F*_(5, 30)_ = 75.86, *P* < 0.0001). **(D,E)** Microbiota community analysis based on PCA and PLS-DA score plots. Data are shown as mean ± SD (*n* = 7 per group). ^****^*P* < 0.0001. PCA, principal component analysis; PLS-DA, partial least squares discriminant analysis.

Supplementary Figure 5 Microbiota community analysis of the donors and recipients. **(A,B)** Microbiota community analysis based on PCA and PLS-DA score plots. Each point represents a sample, and points of the same color belong to the same grouping. Green is the donor L group, dark blue is the donor Z group, red is the recipients L-P group, yellow is the recipients L-Lg group, light blue is the recipients L-Zg group, purple is the recipients Z-P group, orange is the recipients Z-Lg group, and pink is the recipients Z-Zg group. PCA, principal component analysis; PLS-DA, partial least squares discriminant analysis.

Supplementary Figure 6 OPLS-DA after GMT in ZDF recipient rats. **(A)** Diagnostic parameters and regression curves of pairwise comparison between the L-P group and Z-P group. **(B)** Diagnostic parameters and regression curves of pairwise comparison between the Z-P group and Z-Lg group. **(C)** Diagnostic parameters and regression curves of pairwise comparison between the Z-P group and Z-Zg group. OPLS-DA, Orthogonal partial least squares discriminant analysis.
