# Supplementary figures and images for: Integrated Analyses of Microbiomics and Metabolomics Explore the Effect of Gut Microbiota Transplantation on Diabetes-Associated Cognitive Decline in Zucker Diabetic Fatty Rats
Source: Front Aging Neurosci. 2022 Jun 3;14:913002. doi: 10.3389/fnagi.2022.913002 (PMC9204715; doi:10.3389/fnagi.2022.913002)

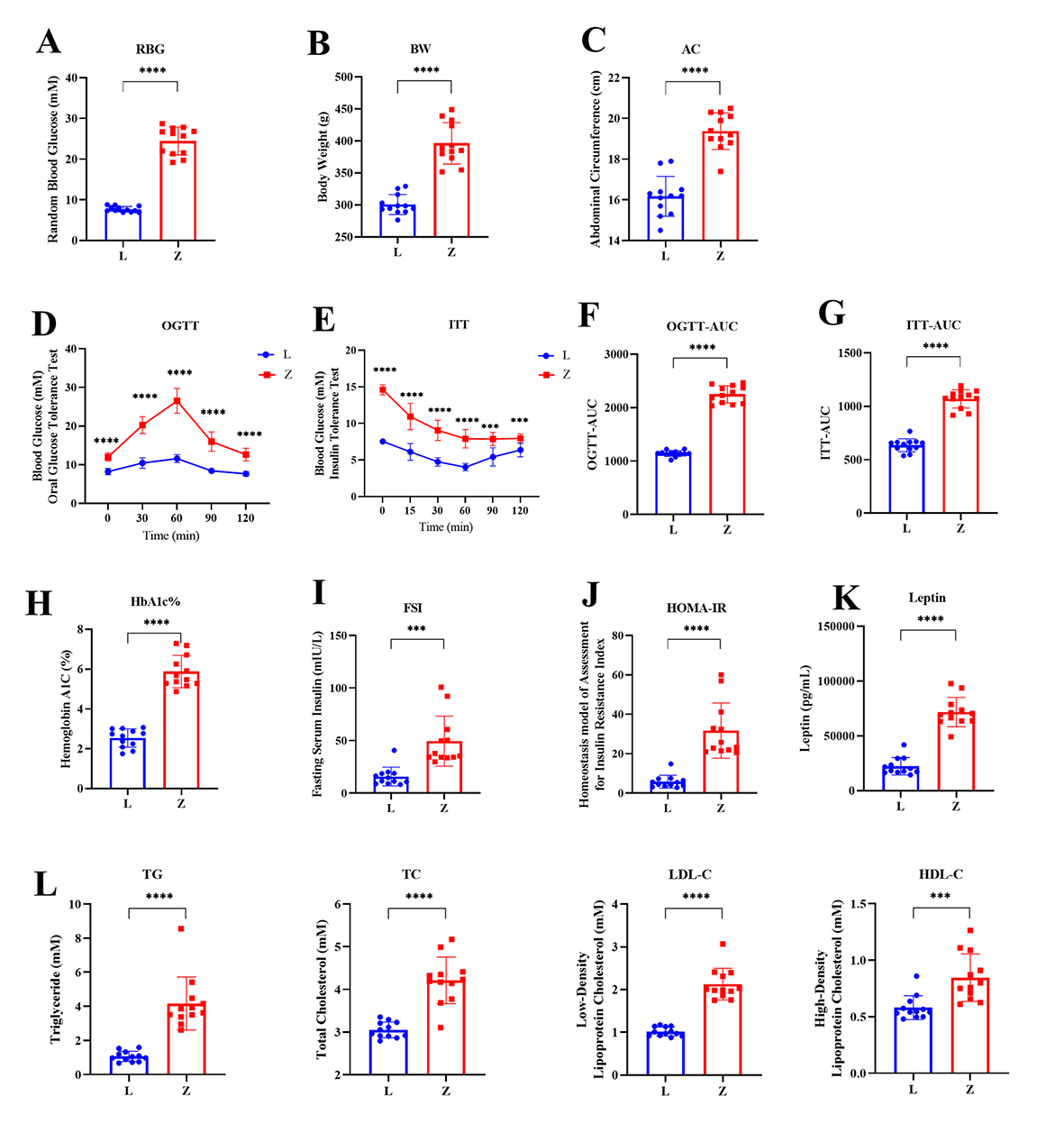

Supplement: Supplementary Figure 1 — Basal metabolism phenotype of the donor rats. (A) Random blood glucose (RBG; t = 16.56, df = 22, P < 0.0001). (B) Body weight (BW; t = 9.203, df = 22, P < 0.0001). (C) Abdominal circumference (AC; t = 8.333, df = 22, P < 0.0001). (D) Oral glucose tolerance test [OGTT; Time: F(4, 88) = 133.1, P < 0.0001; Group: F(1, 22) = 484.1, P < 0.0001]. (E) Insulin secretion test [ITT; Time: F(3.154, 69.38) = 91.55, P < 0.0001; Group: F(1, 22) = 308.6, P < 0.0001]. (F) Area under the curve of the oral glucose tolerance test (OGTT-AUC; t = 22.62, df = 22, P < 0.0001). (G) Area under the curve of the insulin secretion test (ITT-AUC; t = 14.28, df = 22, P < 0.0001). (H) Glycosylated hemoglobin (HbA1c%; t = 12.38, df = 22, P < 0.0001). (I) Fasting serum insulin (FSI; t = 4.581, df = 22, P = 0.0001). (J) Homeostasis model of assessment for insulin resistance index (HOMA-IR; t = 6.246, df = 22, P < 0.0001). (K) Leptin (t = 11.05, df = 22, P < 0.0001). (L) Triglyceride (TG; t = 6.760, df = 22, P < 0.0001), total cholesterol (TC; t = 6.962, df = 22, P < 0.0001), low-density lipoprotein cholesterol (LDL-C; t = 9.992, df = 22, P < 0.0001) and high-density lipid cholesterol (HDL-C; t = 3.918, df = 22, P = 0.0007). Data are shown as mean ± SD (n = 12 per group). ***P<0.001, ****P<0.0001. Two-tailed unpaired Student’s t-test. [file Image_1.TIF]

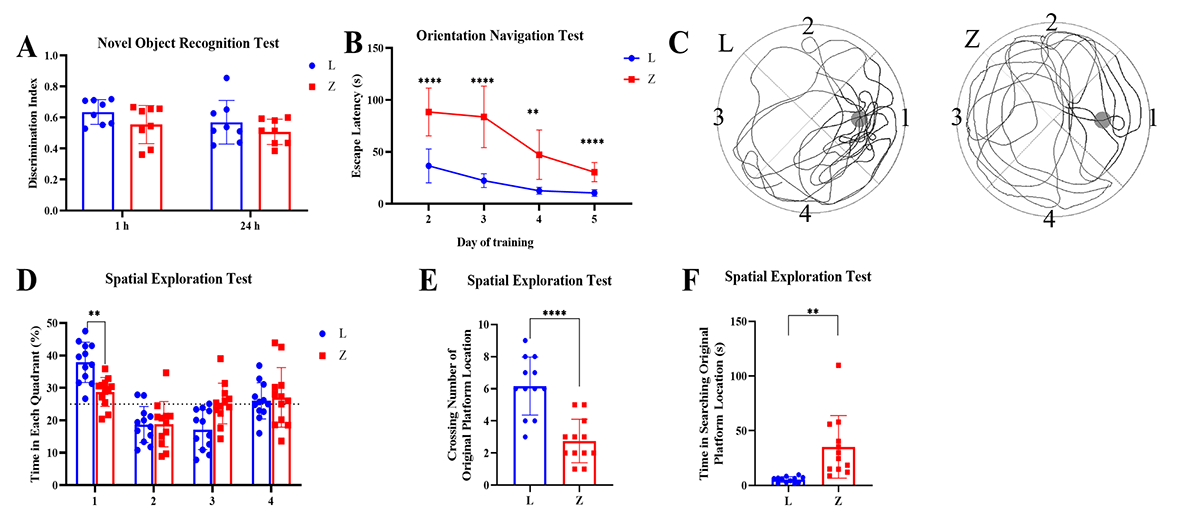

Supplement: Supplementary Figure 2 — Cognitive function of the donor rats. (A) Discrimination index in the new object recognition test (t = 1.083, df = 14, P = 0.2973). (B) The escape latency during a 4 days training course in the orientation navigation test [Time: F(2.348, 51.65) = 38.69, P < 0.0001; Group: F(1, 22) = 87.86, P < 0.0001]. (C–F) Representative swimming path (C), time spent in each quadrant (%) (dotted line indicates that the average chance of entering each quadrant is 25%) (D) (t = 4.127, df = 22, P = 0.0004), crossing number of the original platform location (E) (t = 5.250, df = 22, P < 0.0001) and time in searching original platform location (F) (t = 3.599, df = 22, P = 0.016) in the spatial exploration test. The small circle in Figure 2C represents the original platform location, but the escape platform was removed in the spatial exploration test. Data are shown as mean ± SD (n = 8–12 per group). **P < 0.01, ****P < 0.0001. Two-tailed unpaired Student’s t-test. [file Image_2.TIF]

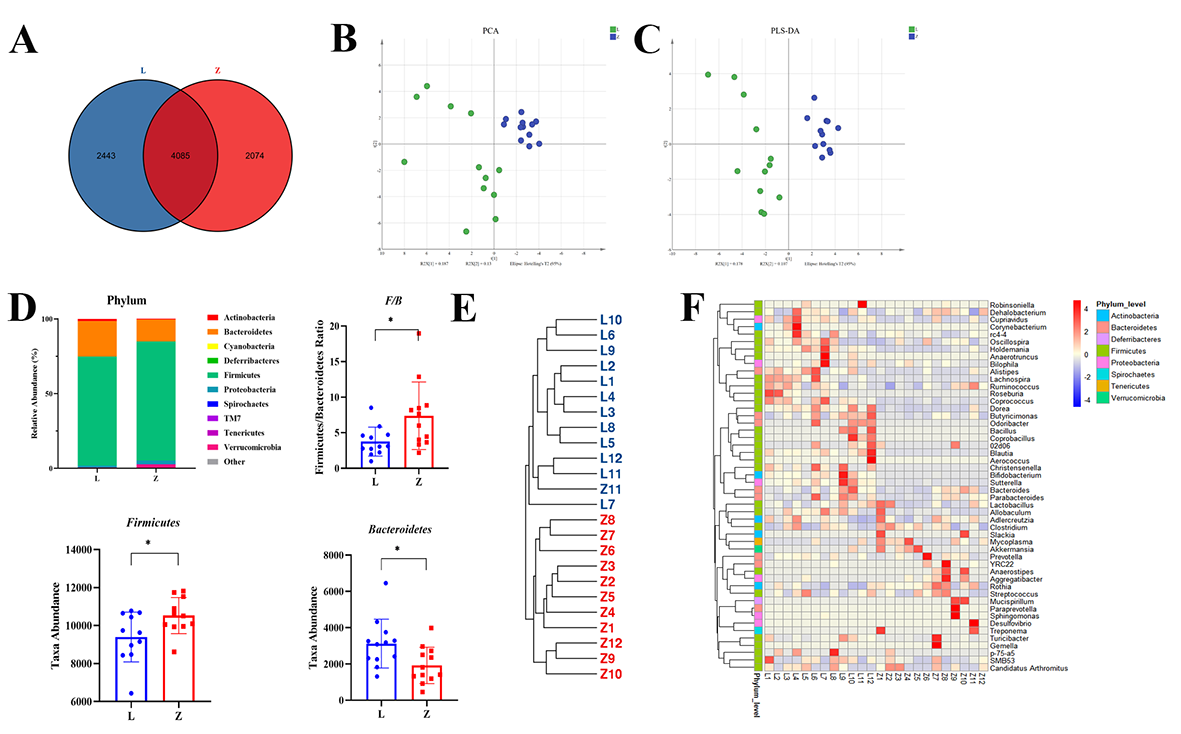

Supplement: Supplementary Figure 3 — Gut microbiota structure of the donor rats. (A) The number of OTUs that are distinct and shared across the groups by Venn diagrams. (B,C) Microbiota community analysis based on PCA and PLS-DA score plots. (D) Bacterial taxonomic composition profiling of gut microbiota at the phylum level (F/B: t = 2.444, df = 22, P = 0.0230; Firmicutes: t = 2.311, df = 20, P = 0.0316; Bacteroidetes: t = 2.483, df = 22, P = 0.0211). (E) Phylogenetic tree map of the bacterial at the genus level. (F) Hierarchical clustering heatmap of the top 50 differentiated taxa at the genus level. Data are shown as mean ± SD (n = 12 per group). *P<0.05. Two-tailed unpaired Student’s t-test. OTUs, operational taxonomic units; PCA, principal component analysis; PLS-DA, partial least squares discriminant analysis. [file Image_3.TIF]

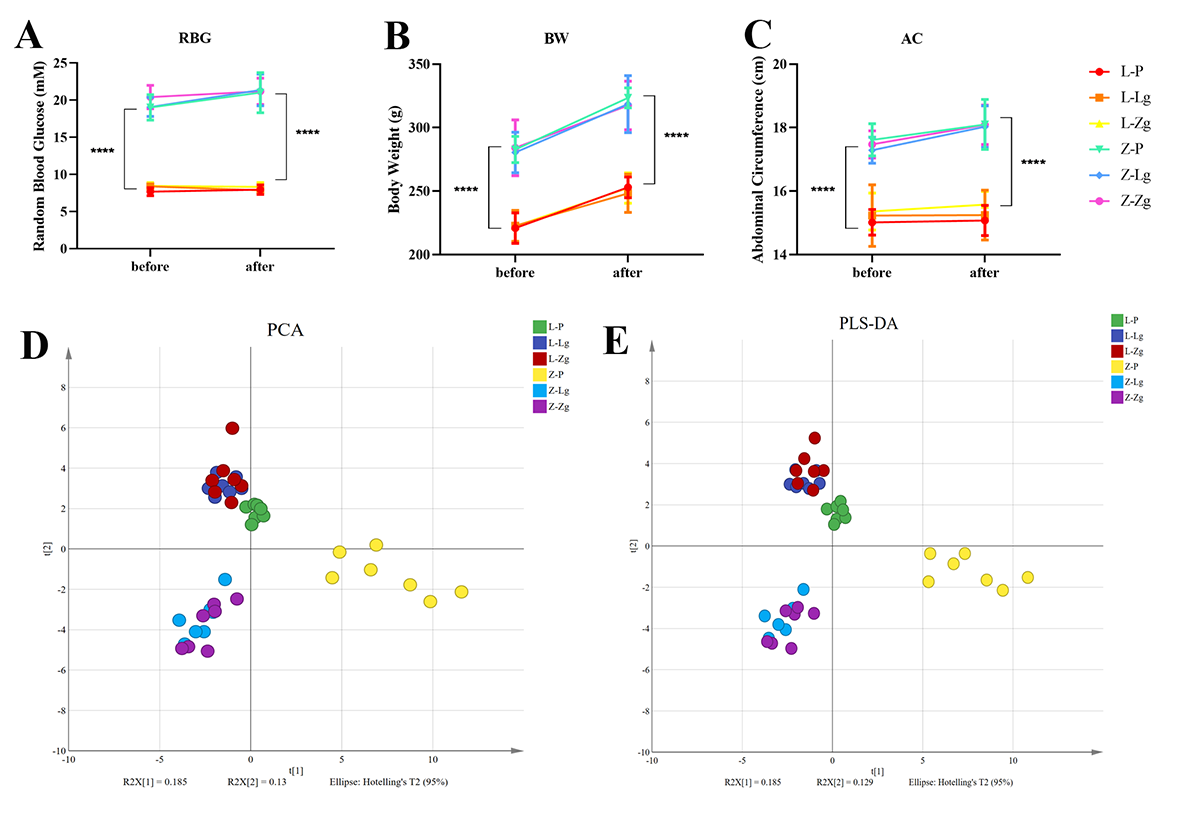

Supplement: Supplementary Figure 4 — Basal phenotype and gut microbiota composition before and after GMT in ZDF recipient rats. (A) Random blood glucose [RBG; Time: F(1, 6) = 9.809, P = 0.0203; Group: F(5, 30) = 207.6, P < 0.0001]. (B) Body weight [BW; Time: F(1, 6) = 285.0, P < 0.0001; Group: F(5, 30) = 40.09, P < 0.0001]. (C) Abdominal circumference (AC; Time: F(1, 6) = 14.23, P = 0.0093; Group: F(5, 30) = 75.86, P < 0.0001). (D,E) Microbiota community analysis based on PCA and PLS-DA score plots. Data are shown as mean ± SD (n = 7 per group). ****P < 0.0001. PCA, principal component analysis; PLS-DA, partial least squares discriminant analysis. [file Image_4.TIF]

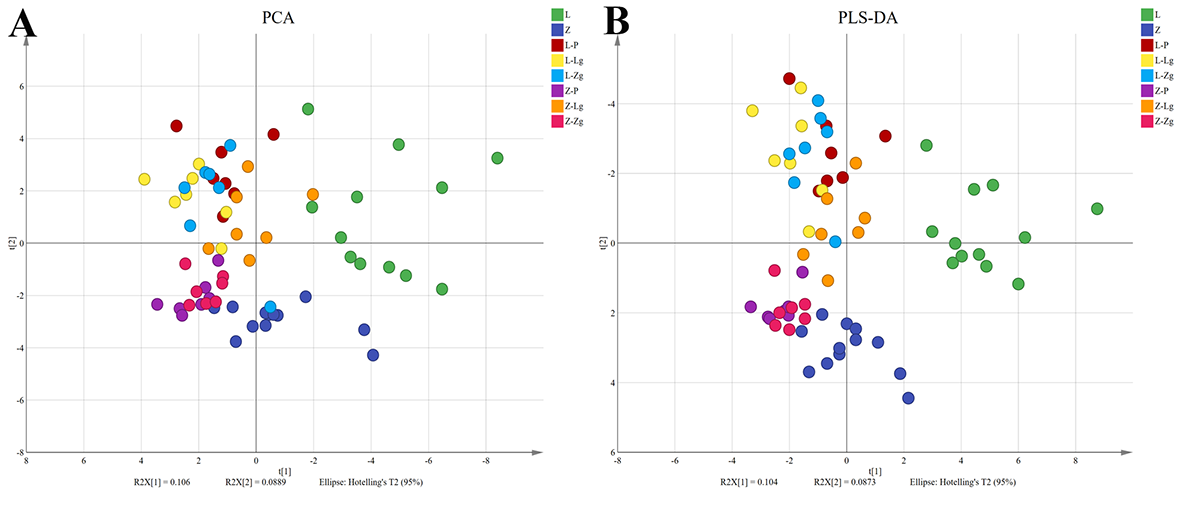

Supplement: Supplementary Figure 5 — Microbiota community analysis of the donors and recipients. (A,B) Microbiota community analysis based on PCA and PLS-DA score plots. Each point represents a sample, and points of the same color belong to the same grouping. Green is the donor L group, dark blue is the donor Z group, red is the recipients L-P group, yellow is the recipients L-Lg group, light blue is the recipients L-Zg group, purple is the recipients Z-P group, orange is the recipients Z-Lg group, and pink is the recipients Z-Zg group. PCA, principal component analysis; PLS-DA, partial least squares discriminant analysis. [file Image_5.TIF]

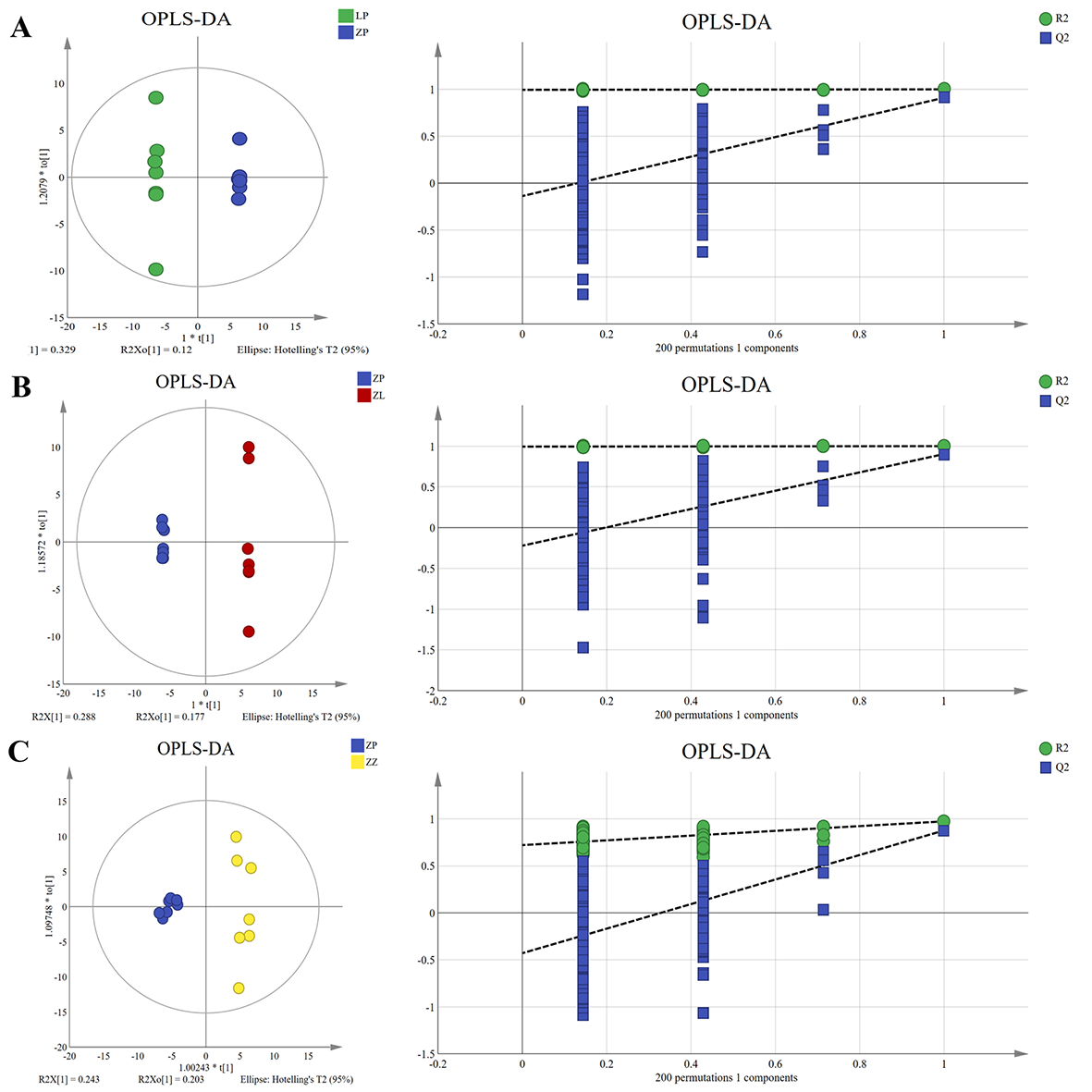

Supplement: Supplementary Figure 6 — OPLS-DA after GMT in ZDF recipient rats. (A) Diagnostic parameters and regression curves of pairwise comparison between the L-P group and Z-P group. (B) Diagnostic parameters and regression curves of pairwise comparison between the Z-P group and Z-Lg group. (C) Diagnostic parameters and regression curves of pairwise comparison between the Z-P group and Z-Zg group. OPLS-DA, Orthogonal partial least squares discriminant analysis. [file Image_6.TIF]
